# Effectiveness of probiotics and clove essential oils in improving growth performance, immuno-antioxidant status, ileum morphometric, and microbial community structure for heat-stressed broilers

**DOI:** 10.1038/s41598-023-45868-9

**Published:** 2023-11-01

**Authors:** Ahmed M. Elbaz, Eman S. Ashmawy, Safaa A. M. Ali, Disouky M. Mourad, Hanan S. El-Samahy, Faisal B. Badri, Hany A. Thabet

**Affiliations:** 1 Poultry Nutrition Department, Desert Research Center, Mataria, Cairo, Egypt; 2 Poultry Physiological Department, Desert Research Center, Mataria, Cairo, Egypt; 3 Animal and Poultry Health Department, Desert Research Center, Mataria, Cairo, Egypt; 4 Poultry Production Department, Faculty of Agriculture, Ain Shams University, Cairo, Egypt

**Keywords:** Immunology, Microbiology, Animal physiology

## Abstract

Recently, interest has increased in using bio-additives, herbs, and their extracts as feed additives because of their potential role in improving chick's health and productivity, especially during stress. Thus, our aim in this study is to examine whether nutritional supplementation (probiotics and clove essential oils) will help mitigate the negative effect of heat stress on the bird by modifying the microbial content, boosting immunity, oxidative status, metabolic, and growth. In this study, three hundred one-day-old broiler chicks (Ross 308) were fed the following experimental diet: (CON) basal diet (control diet); (CEO) CON with clove essential oils (300 mg/kg); (PRO) CON with probiotics (2 g/kg); (PC) CON with probiotics and clove essential oils. Our results showed a significant improvement (*P* < 0.05) in body weight gain, feed conversion ratio, nutrient digestibility, and digestive enzymes activities in broilers fed on PC, CEO, and PRO compared to the control group. Moreover, a significant decrease was recorded in the abdominal fat content and an increase in the relative weight of bursa of Fabricius, and higher antibody levels against Newcastle disease virus, as well as, there was an increase (*P* < 0.05) in interleukin 10 (IL-10) in all treated groups. Meanwhile, there was a decrease in tumor necrosis factor-α (TNF-α) in all supplemented groups compared with the control group. Serum triglycerides, cholesterol, low-density lipoprotein concentrations, and alanine aminotransferase activities were significantly lower in the treated groups. Superoxide dismutase and glutathione peroxidase levels were elevated (*P* < 0.05) and the malondialdehyde level value significantly decreased in all supplemented groups. The treated groups enhanced the ileum structure by increasing *Lactobacillus*, decreasing *E. coli*, and improving the morphometrically (*P* < 0.05). This study strongly suggests that clove essential oil and probiotic mixture can be used as a feed supplement to reduce the effects of heat stress by improving the growth performance and enhancing immuno-antioxidant status, ileum morphometric, as well as modifying the microbial community structure of the ileum of broilers.

## Introduction

High temperature represents a major threat to animal production, especially poultry. Furthermore, the genetic developments in broiler chick strains to increase the growth rate made them more vulnerable to environmental stress. The high sensitivity to environmental stress results from the inferior development of the respiratory system and the lack of sweat glands in poultry^1^. Moreover, the large consumption of feed leads to high thermal output (Metabolic heat). When a bird is exposed to high temperatures, the body begins to work on losing excess heat to mitigate the effects of heat stress^2^. In other words, the bird's ability to lose internal heat reduces, leading the bird to enter a stage of danger^3^. The heat stress leads to a series of changes in the bird's behavior, such as evaporative heat loss (panting), reduced feed consumption, increase drinking water, perturbations in blood pH, changes in gut microbial, inflammation in the intestinal wall, loss of immunity, and decreasing growth rate and survival rate^4,5^. Which made nutrition experts search for feed additives to enhance bird performance during stress. Many studies have recommended adding some nutritional additives to reduce the harmful effects of heat stress, such as vitamins, organic acids, spices, herbs, probiotics, etc.^6,7^.

Probiotics are beneficial organisms that are added to enhance growth, modify the gut microbiome, and stimulate immunity^8^. In addition to reducing the harmful effects of heat stress by protecting the intestines from disease-causing microbes, and promoting the digestion of nutrients^9^. Herbs and their extracts (essential oils) are used for many medicinal purposes as feed additives in animal diets to improve general health. Furthermore, it improves feed intake and flavor, increasing gastric and intestinal motility, stimulating the secretion of digestive enzymes^10^, antimicrobial^11^, antioxidative activity^12^, immune stimulation, and anti-inflammatory^13^. Clove and its extracts (*Syzygium aromaticum*) are among the most used medicinal herbs for their high content of biologically active compounds that have antimicrobial and antioxidant properties. Moreover, it stimulates growth, immunity, and hypochelesterolemic activities^14^, which is due to its antimicrobial, antioxidant, and digestive properties^13^. We hypothesized that clove essential oils and probiotics combined might alleviate the adverse effects of heat stress on growth performance, by enhancing immuno-antioxidant status, and intestinal health.

## Results

### Growth performance and carcass traits

Growth performance improved in broilers fed a diet containing probiotics, clove essential oil, or their mixture as shown in Table 1. PRO and PC groups recorded a higher (*P* < 0.05) BWG than CEO and CON groups during the starter period, while PC and CEO groups recorded higher (*P* < 0.05) BWG than PRO and CON groups during the overall experimental periods. Feed intake was not affected by the experimental treatments during the different experimental periods. Overall, FCR improved (*P* < 0.05) in all treated groups compared to the untreated one (CON). Broilers fed the combination of clove essential oil and probiotics (PC) had the best FCR in the overall experimental period. The best growth performance was in broilers fed PC, it had the highest BWG and a better FCR. Furthermore, there was no effect of dietary treatments on carcass yield, dressing percentages, and the liver relative weight as shown in Table 1. While birds fed on dietary treatments showed a significant decrease (*P* < 0.05) in abdominal fat compared with the control group, broilers fed CEO had the lowest values of abdominal fat.

**Table 1:** Effect of supplementation of probiotics and clove essential oil on growth performance and carcass traits of heat-stressed broiler chickens.

Parameter	CON	PRO	CEO	PC	SEM	*P* value
Starter (1–20 d)						
BWG (g)	748^c^	803^a^	776^b^	811^a^	15.06	0.031
FI (g)	951	972	956	976	21.44	0.377
FCR (g:g)	1.270^a^	1.210^b^	1.232^ab^	1.205^b^	0.072	0.016
Grower (21–35 d)						
BWG (g)	902^b^	942a^b^	1004^a^	983^a^	23.19	0.020
FI (g)	2082	2090	2176	2108	40.33	0.075
FCR (g:g)	2.308^a^	2.219^b^	2.168^c^	2.145^c^	0.091	< 0.001
Overall (1–35 d)						
BWG (g)	1651^c^	1746^b^	1780^a^	1794^a^	38.54	0.018
FI (g)	3032	3062	3131	3083	51.08	0.093
FCR (g:g)	1.837^a^	1.755^b^	1.761^b^	1.720^c^	0.063	< 0.001
Carcass traits (%)						
Dressing	70.3	71.4	70.9	71.2	0.224	0.187
Carcass yield	72.6	74.2	73.4	73.1	0.109	0.095
Liver	1.67	1.71	1.63	1.75	0.069	0.119
Abdominal fat	7.29^a^	6.35^b^	7.16^a^	6.24^b^	0.118	0.001

*BWG* body weight gain, *FI* feed intake, *FCR* feed conversion ratio, *CON* basal diet without added, *PRO* basal diet with probiotics, *CEO* basal diet with clove essential oil, *PC* basal diet with probiotics and clove essential oil.

^a,b,c^Means in the same row with different superscripts are significantly different significantly (*P* < 0.05).

### Nutrient digestibility and digestive enzymes

Our results showed a significant effect of dietary treatments on nutrient digestibility, as shown in Table 2. The control group had a reduced nutrient digestibility compared to the other groups (*P* < 0.05) under heat stress. PC and PRO groups had higher (*P* < 0.05) DM and CP compared with the control and CEO groups. Similarly, the PC and PRO groups had higher (*P* < 0.05) EE. Probiotics or their mixture with clove essential oil improved DM, CP, and EE digestibility (*P* < 0.05). Interestingly, our results showed a significant effect of dietary treatments on the activity of digestive enzymes except for trypsin, as shown in Table 2. The addition of probiotics, clove essential oil, or combined increased the lipase enzyme activity compared to the control group. Adding clove essential oil (CEO) or its mixture with probiotics (PC) to the diet led to an increase in amylase enzyme activity compared with the PRO and control groups.

**Table 2:** Effect of supplementation of probiotics and clove essential oil on nutrient digestibility (%) and digestive enzymes activities (U/g) of heat-stressed broiler chickens.

Parameter	CON	PRO	CEO	PC	SEM	p-value
Nutrient digestibility						
Dry matter	68.4^b^	73.5^a^	70.3^ab^	74.1^a^	1.851	0.040
Crude protein	71.7^b^	74.2^a^	72.0^b^	75.6^a^	4.772	0.015
Ether extracts	64.9^b^	69.1^a^	65.4^b^	70.2^a^	0.988	0.011
Digestive enzymes						
Amylase	157^b^	163^b^	182^a^	179^a^	24.57	0.016
Trypsin	94.6	95.4	97.5	96.2	12.48	0.122
Lipase	67.5^c^	71.8^bc^	75.1^b^	80.7^a^	18.12	< 0.001

*CON* basal diet without added, *PRO* basal diet with probiotics, *CEO* basal diet with clove essential oil, *PC* basal diet with probiotics and clove essential oil.

^a,b,c^ Means in the same row with different superscripts are significantly different significantly (*P* < 0.05).

### Serum chemistry and immune responses

The effect of dietary treatments on serum lipid profile, hepatic biomarkers, and antioxidative enzymes activities of heat-stressed broilers at day 35 are shown in Table 3. There was a significant decrease in serum MDA levels, while serum SOD and GPx levels were increased (*P* < 0.05) in the PC, PRO, and CEO groups. Serum lipid profile was greatly affected by dietary supplements; triglycerides, cholesterol, and LDL decreased (*P* < 0.05), while HDL was not affected (*P* < 0.05). No significant change in serum levels of glucose, total proteins, and albumin among experimental groups. ALT serum level (an indicator of hepatic health) significantly decreased in all treated groups compared to the control group, while AST serum level was not affected (*P* < 0.05).

**Table 3:** Effect of supplementation of probiotics and clove essential oil on serum lipid profile, hepatic biomarkers, and antioxidative enzymes activities of heat-stressed broiler chickens.

Parameter	CON	PRO	CEO	PC	SEM	*P* value
Lipid profile						
Glucose (mg dl^−1^)	71.2	72.4	70.5	68.6	0.950	0.078
Triglycerides (mg dl^−1^)	227.1^a^	187.6^c^	207.2^b^	197.5^bc^	2.084	0.001
Cholesterol (mg dl^−1^)	219.5^a^	199.0^bc^	186.6^b^	173.1^c^	1.447	< 0.001
HDL (mg dl^−1^)	61.7	70.3	66.5	68.9	3.081	0.009
LDL (mg dl^−1^)	121.5^a^	89.4^cd^	98.3^b^	83.1^d^	1.095	0.015
Hepatic biomarkers						
Total Proteins (g dl^−1^)	3.29	3.34	3.23	3.19	0.834	0.107
Albumin (g dl^−1^)	1.24	1.30	1.27	1.29	0.311	0.091
AST (U L^−1^)	81.6	79.1	82.0	80.6	0.507	0.083
ALT (U L^−1^)	27.5^a^	23.2^cd^	25.4^ab^	24.4^c^	0.442	0.017
Antioxidative enzymes						
MDA (nmol ml-1)	1.381^a^	1.072^b^	0.959^bc^	0.861^c^	0.914	< 0.001
SOD (U ml-1)	116.4^d^	133.2^a^	125.4^bc^	128.7^b^	2.087	< 0.001
GPx (U ml-1)	14.52^d^	28.57^b^	21.64^c^	30.90^a^	1.125	< 0.001

*CON* basal diet without added, *PRO* basal diet with probiotics, *CEO* basal diet with clove essential oil, *PC* basal diet with probiotics and clove essential oil, *HDL* high-density lipoprotein, *LDL* low-density lipoprotein, *AST* aspartate aminotransferase, *ALT* alanine aminotransferase, *MDA* malondialdehyde, *SOD* superoxide dismutase, *GPx* glutathione peroxidase.

^a,b,c^Means in the same row with different superscripts are significantly different significantly (*P* < 0.05).

The effect of dietary treatments on immune organs’ relative weight and humoral immune response of heat-stressed broilers at day 35 are shown in Table 4. Dietary supplements had no significant effect on the thymus and spleen relative weight, while the bursa of Fabricius relative weight increased (*P* < 0.05) in the PC, CEO, and PRO groups. Broilers fed PC, CEO, and PRO under heat stress had the highest antibody levels against NDV, while serum antibody titer against AIV did not significantly (*P* < 0.05) change among experimental groups. Moreover, interleukin 10 (IL-10) increased, while tumor necrosis factor-α (TNF-α) concentrations decreased (*P* < 0.05) in broilers fed PC, CEO, and PRO compared with the control group. Experimental supplementation did not influence serum interleukin 6 (IL-6) concentrations (*P* < 0.05). Feeding a diet containing both probiotics and essential oils together (PC group) had the best anti-inflammatory effect.

**Table 4:** Effect of supplementation of probiotics and clove essential oil on immune responses of heat-stressed broiler chickens.

Parameter	CON	PRO	CEO	PC	SEM	*P* value
Immune organs (%)						
Thymus	0.180	0.191	0.184	0.181	0.017	0.251
Spleen	0.163	0.173	0.168	0.175	0.009	0.124
Bursa of Fabricius	0.148^d^	0.316^ab^	0.207^c^	0.374^a^	0.041	0.003
Antibody titer against						
AIV	3.24	3.49	3.31	3.42	0.110	0.211
NDV	4.31^c^	5.54^a^	5.12^b^	5.67^a^	0.049	< 0.001
Immune cytokines						
IL-10 (pg/mL)	34.9^b^	39.0^a^	37.2^ab^	40.5^a^	0.380	0.014
IL-6 (pg/mL)	87.5	83.1	82.9	85.6	0.559	0.072
TNF-α (pg/mL)	257^a^	242^ab^	236^b^	221^c^	1.071	< 0.001

*CON* basal diet without added, *PRO* basal diet with probiotics, *CEO* basal diet with clove essential oil, *PC* basal diet with probiotics and clove essential oil, *AIV* avian influenza virus, *NDV* Newcastle disease, *IL-10* cytokines of interleukin-10, *IL-6* cytokines of interleukin-6, *TNF-α* tumor necrosis factor-α.

^a,b,c^Means in the same row with different superscripts are significantly different significantly (*P* < 0.05).

### Ileal microbial and morphometric changes

The results presented in Table 5 show the effect of dietary treatments on the ileum microbial composition of broiler chicken under heat stress. Diet supplements, either combined or individual, had beneficial effects on the ileum microbial content. The *Lactobacillus* population increased significantly and the *E. coli* population decreased significantly (*P* < 0.05) in chickens fed a diet containing the experimental supplements. PC and PRO groups showed a significant increase in the count of *Lactobacillus* compared to the other groups. Furthermore, there was a significant decrease (*P* < 0.05) in the *E. coli* population in the ileum in PC and PRO groups followed by the CEO group compared to the control group. Meanwhile, the experimental treatments did not affect the count of total coliforms and *Enterococcus* in the ileum. However, there was a numerical decrease (*P* = 0.063) in the count of total coliforms of broilers that received PRO, PC, and CEO compared to broilers that received CON.

**Table 5:** Effect of supplementation of probiotics and clove essential oil on ileal microbial enumeration (Log10 CFU g^−1^) and histomorphological parameters (μm) of heat-stressed broiler chickens.

Parameter	CON	PRO	CEO	PC	SEM	*P* value
Microbial enumeration						
*Lactobacillus*	4.157d	5.044b	4.621c	5.583a	0.270	< 0.001
*Escherichia coli*	5.341^a^	4.282^bc^	4.705^b^	3.837^d^	0.099	< 0.001
*Enterococcus*	5.720	5.374	5.551	5.704	0.519	0.086
Total coliforms	3.281	2.931	3.072	2.875	0.281	0.063
Histomorphological						
Villus height (VH)	1168^d^	1411^b^	1366^c^	1759^a^	5.068	< 0.001
Crypt depth (CD)	204^b^	216^a^	207^b^	219^a^	2.733	0.020
VH/CD	5.74^c^	6.53^b^	6.46^b^	8.02^a^	0.370	0.018

*CON* basal diet without added, *PRO* basal diet with probiotics, *CEO* basal diet with clove essential oil, *PC* basal diet with probiotics and clove essential oil, *VH/CD* Villus height/Crypt depth.

^a,b,c^Means in the same row with different superscripts are significantly different significantly (*P* < 0.05).

The data presented in Table 5 show that all the experimental treatments had a significant improvement in the ileal villus height (VH) compared to the control group. Chickens fed on PC had the highest VH compared to the other experimental groups (*P* < 0.05). Moreover, the chickens fed the PC and PRO showed the highest value of crypt depth (CD) compared to the other groups. Whilst the villus height: crypt depth ratio in the PC group increased (*P* < 0.05) more than in the other groups, there was a significant increase in the VH: CD ratio in the PRO and CEO groups in comparison with the control group.

## Discussion

Several previous studies confirmed that the bird's exposure to heat stress causes great economic loss through high mortality and poor growth performance^4,5^. The most important reasons for the decline in performance during the period of heat stress are the reduction in the feed intake, which is caused by the influence of the peripheral thermal receptors that transmit nerve impulses that suppress the activity of the appetite center in the hypothalamus^15^, likewise the imbalance of the microbial system of the digestive system, which reduces the utilization of nutrients. Furthermore, heat stress increases the risk of oxidative stress exposure. Previous studies confirmed that the use of some nutritional supplements achieved mitigating the negative effects of heat stress^6,12,16^. The results of the current study indicated that the mixture of probiotics and EO significantly improved the performance of heat-stressed broiler chickens. In general, the use of probiotics and clove essential oils as an additive showed a significant improvement in body weight gain and feed conversion ratio compared to the control group. Our current results are consistent with Attia et al.^13^; Elbaz et al.^17^; and Yin et al.^18^ who reported that including EO and probiotics as a dietary supplement improved productive performance in terms of live body weight and feed conversion rate. Similar to our findings, Attia et al.^13^ have reported that the dietary addition of EO enhanced growth performance in birds during summer. The improved growth performance may be due to the high potential of combining probiotics with EO which enhances feed utilization and general health, including the stimulating effect on the activity of digestive enzymes, antimicrobial, antioxidant, and immunity^19,20^. In addition, broilers fed a diet containing EO and probiotics showed a significant improvement in growth performance, which may be due to the synergistic effect of probiotics and EO on enhancing gut health and improving digestion and absorption of nutrients, in addition to the properties of phenolic compounds that alleviate the thermal stress effect.

It is interesting that with the noticeable improvement in growth performance in our results, there was no effect of the dietary supplement on the carcass dressing and carcass yield, meanwhile, the addition of probiotics reduced abdominal fat content. However, there was an insignificant numerical increase in the carcass yield and dressing, which may be due to the improvement in the digestion and absorption of nutrients^20,21^. Our results were supported by many studies, which indicate that the carcass percentage and the relative weight of the internal organs of broiler chickens fed a diet that includes EO or probiotics were not affected^22,23^. Several studies have also confirmed that adding live microbes to a broiler diet reduces the carcass content of abdominal fat^24,25^. This can be explained by the effect of adding probiotics on the reduction of the acetyl-CoA carboxylase enzyme, which has a catalytic role in the synthesis of fatty acids, which reduces lipogenesis^26,27^. Low abdominal fat represents a great economic benefit in the poultry industry. Abdominal fat is the main source of waste (excess fats) in the slaughterhouse^28,29^, therefore, the low content of abdominal fat in the carcass leads to an increased carcass yield, which increases the total productivity of the slaughterhouse. The lack of abdominal fat indicates the beneficial effect of the experimental dietary supplement on the distribution of fat in the carcass, where the utilized distribution of the fatty acids between the muscles contributes to improving carcass quality^30^. However, there is still a need for more studies on the fatty acid composition of carcass meat to determine the meat quality.

Previous studies documented the beneficial effects of probiotics and essential oils on the nutrient digestibility of broiler chickens^8,31^. Similarly, the current study indicated that the addition of probiotics or EO or both had a positive effect on the nutrient digestion of heat-stressed broiler chickens. The remarkable improvement in nutrient digestion can be explained by the effect of probiotics on the modification of the microbial content in the intestine^32^ and the reduction of toxic compounds resulting from pathogenic microbes, in addition to the breakdown of complex food compounds into simple compounds which improve its utilization^33^. Alloui et al.^34^ reported the positive impact of EO-based products on stimulating the secretion of mucus, and saliva and the activities of the digestive enzymes. The addition of EO improved the digestion of nutrients, which is due to the improvement of the microbial balance of the digestive system, thus improving the digestion and absorption of nutrients^10^, which is confirmed by our following results. The results showed a higher amylase activity in chickens fed PC and CEO. Furthermore, the activity of the lipase enzyme was higher in chickens fed PC, CEO, and PRO, while trypsin enzyme activity was not affected by experimental treatments. Previous studies confirmed that adding EO or probiotics to the diet stimulates digestive enzymes activity and increased bile acids^35,36^, which enhanced nutrient digestion and improved the feed conversion ratio, thus improving the growth performance of heat-stressed broilers.

The bird’s exposure to heat stress leads to a reduction in feed intake, that's when the body starts using stored energy instead of digesting to reduce the amount of energy produced during heat stress, which affects the work of vital organs in the body and thus leading to significant physiological changes. The liver is one of the most important vital organs and the most affected by nutritional changes, which affect the lipid profile, and antioxidants, in addition to detoxification. Interestingly, despite the changes in serum lipid profile and antioxidant status with experimental dietary additions, the relative weight of the liver was not affected in this study. Our results indicated that feeding a diet supplemented with probiotic or clove essential oil significantly decreased serum triglycerides, cholesterol, LDL, and ALT compared to broilers fed a control diet during the heat stress period. Similar to those reported by Kirkpinar et al.^37^ where the addition of oregano in broiler diets reduced cholesterol and triglycerides compared to the control group. Results obtained in this study are in agreement with results reported by Ali et al.^38^ who found that adding thyme to hen diets significantly decreased blood total cholesterol and triglycerides. The hypocholesterolemic in the blood may be due to some components of essential oils that inhibit 3-hydroxy-3-methylglutaryl coenzyme A reductase, which is the enzyme responsible for cholesterol synthesis^39^. It has been reported that probiotics and EO significantly decrease serum LDL levels^40,41^. In this study, adding probiotics and essential oil led to a significant decrease in these parameters. Similar to previous studies, our results showed that serum total protein and albumin were not affected by dietary supplements^41,42^. Furthermore, our results have similarities with Santoso et al.^43^ who reported that adding probiotics lowered levels of ALT enzyme compared with the control treatment.

The use of natural antioxidants in poultry feed is an important means of controlling fat oxidation. Numerous studies have confirmed the distinctive role of EO as a natural antioxidant^44^. It was proven that the use of natural plants and their derivatives promoted oxidative stability inside the bird's body^45^. Bird exposure to heat stress leads to an imbalance between the biological antioxidant system and the rate of producing free radicals in the body, which is known as oxidative stress^46^. In this study, certain dietary supplements were used to evaluate their ability to enhance the antioxidant status of heat-stressed broiler chickens. Our results confirmed that the addition of probiotics and EO had a role in enhancing the oxidative state of heat-stressed chickens. The results of the current study indicated that broilers fed the mixture of probiotics and clove essential oil had the lowest level of MDA and the highest SOD and GPx in serum than the control. Our current results agreed with Guldiken et al.^47^, who found that the addition of EO (clove, rosemary, and thyme) enhanced the activity of antioxidants. Previous studies reported that the addition of essential oils improved the antioxidant capacity of heat-stressed chickens^12,48^. According to our results, adding both essential oils and probiotics synergistically enhanced the antioxidant status during heat stress, as documented by other studies^17,49^.

Data showed that antibody responses to NDV improved through the experimental treatments. Furthermore, the relative weight of the bursa of Fabricius increased significantly in the PRO and PC groups. The same results were obtained by other studies^17,50^. A study reported that probiotics stimulated sets of immune system cells to produce cytokines, which in turn play a role in the stimulation and regulation of immune response^51^. Elbaz et al.^12^ and Hashemi et al.^52^ reported a significantly higher antibody production in broilers fed probiotics or EO than those fed on control, confirming that this dietary supplementation enhances the humoral response. On the contrary, Azadegan et al.^53^ found that essential oils have no impact on antibody response to NDV and AIV. The inconsistencies in the studies results are a consequence of many factors, such as the type of plant used, harvest time, their physical properties, and herbal extraction methods. Previous studies by Barbour et al.^54^; Gopi et al.^49^ stated that EOs play an important role in alleviating stress resulting from vaccination by enhancing the production of antibodies, thus enhancing the efficacy of vaccination. Numerous studies indicated a connection between the addition of essential oils to chicken's diet and the improvement in cell-mediated, humoral immune response, which increases antibody production^43,55^. It can be concluded that the mixture of EOs and probiotics enhanced the immune response.

The control of intestinal inflammation during stress is the key to healthy birds. Numerous studies show that broiler chickens are at risk of acute infections when exposed to heat stress, which leads to changes in cytokines synthesis such as the increase in tumor necrosis factor-α (TNF-α) and interleukin IL-4 concentrations or the decrease in the concentration of IL-2 in the spleen^56^. Therefore, we estimated the concentration of cytokine and TNF-α (as a detector of inflammation) to reveal the effect of feed experimental supplementation on reducing inflammation resulting from heat stress in this study. The results of the current study showed that compared with the control group, the mixture of probiotics and essential oils reduced inflammation by reducing TNF-α and increasing IL-10 concentrations in serum. TNF-α is a pro-inflammatory cytokine that is secreted by monocytes and macrophages, which is the main indicator of the bird's exposure to stress. On the contrary, IL-10 is a key cytokine to restrain the excessive production of pro-inflammatory cytokines, which are secreted by activated macrophages and maintain the immune balance of birds. Similar to our findings, serum TNF- α concentration decreased in broilers fed probiotic supplementation of *Bacillus licheniformis* during HS^57^. Consistent with our results, dietary yeast hydrolysate supplementation decreased serum TNF-α concentrations and increased IL-10^58,59^. In addition, Ocaña and Reglero^60^ emphasized that thyme oil extracts resulted in an overall reduction of proinflammatory cytokines release, TNFα, IL1β, and IL-6. Administration of cinnamon extract in broilers' diet significantly reduced the tissue levels of TNF-α gene expressions of broiler chickens inoculated with *E. coli* in comparison with the *E. coli* group^61^. It can be concluded that adding probiotics, essential oils, or both reduced inflammations in broilers reared under heat stress.

It is considered to the maintenance of intestinal integrity and function is an important factor for the optimum performance and best health of broiler chickens. The intestinal health of poultry is directly linked with gut function and nutrient digestion and absorption, as well as, immune responses. The extent of intestinal health is determined by indicators such as microbial content and morphological measures. Also, many previous reports confirmed that the use of feed additives had a beneficial effect on intestinal health. In the current study, the use of dietary supplements showed antimicrobial activity, in terms of a significant decrease in *E. coli* counts and an increase in *Lactobacillus* in the ileum of heat-stressed broilers. Essential oil supplementation in broiler diets stabilizes the microflora environment of the avian gut^62^. Furthermore, adding essential oil increases the number of *Lactobacillus* in the ileum^63^. Grimes et al.^11^; Elbaz^23^ reported that supplementing the diet with probiotics results in lowering many enteric pathogens. According to these results, both essential oils and probiotics (PC group) improved intestinal microbial community structure. The improvement in the intestinal microbial content may be due to the biologically active compounds (phenolic compounds) in the essential oils, which lowers the pH of the gastrointestinal tract, thereby inhibiting pathogenic microbes^64^, which was confirmed by the results of the current study. Moreover, one of the most important activities of probiotic microbes is producing organic acids, which lowers the pH in the intestines, giving the beneficial microbes a chance in competing for food and the surface of epithelial cells^65^. This explains the positive correlation between significant improvement of intestinal microbial content and dietary supplementation of heat-stressed broiler chickens in this study.

The changes in the ileum morphometric indicate the potential benefits of dietary supplements on the digestive system, which is reflected in the performance of heat-stressed broilers. Our results recorded a significant increase in the ileal villi height and crypt depth in the PC and PRO groups. These results are in agreement with several studies^12,24,43^. Previous results indicate a direct correlation between the increase in the villi height and the improvement in growth performance by increasing the surface area, which increases the ability to absorb nutrients leading to an enhancement of FCR and BWG^66,67^. Likewise, a study by Viveros et al.^68^ explained that the increase in villus height is associated with the increased expression of brush border enzymes. From this, it can be concluded that the combination of clove essential oils and probiotics had a positive effect on improving the gut health of the broilers exposed to heat stress.

## Conclusion

This study demonstrated that dietary supplementation of clove essential oils, probiotics, or their combinations improved the growth performance and enhanced gut health of heat-stressed broiler chickens. The detrimental impacts of heat stress on growth performance, nutrient digestibility, immuno-antioxidant status, ileal bacterial community structure, and ileal morphometric, were significantly lower in broilers fed on these dietary supplements. As well, a synergistic potential of clove essential oils with probiotics was found to exhibit a greater impact on nutrient digestibility, immune response, and gut health, which improved the productive performance of heat-stressed broilers.

## Materials and methods

### Chicks and experimental design

Three hundred one-day-old (Ross 308) unsexed broiler chicks were purchased from a commercial hatchery, the average weight was 41.3 g. They were randomly distributed into four groups; each group contained 75 chicks (five replicates) in cages (120 × 60 × 50 cm). The experimental groups were as follows: (CON) a basal diet without additives, (PRO) a basal diet with probiotics (2 g/kg diet), (CEO) a basal diet with clove essential oil (300 mg/kg diet), and (PC) a basal diet with probiotics and clove essential oil. The birds were fed on two main diets divided into two stages as follows: the first diet contains 3000 kcal and 23% protein (starter, 1 to 20 days), and the second diet contains 3200 kcal and 21% protein (grower, 21–35 days), feed formulated according to the needs of birds in NRC^69^, as shown in Table 6. Feed and water were left in front of the birds continuously throughout the experiment period. The broiler chicks were subjected contentiously to light for the first 3 days, then light hours were reduced to 22 h a day until 35 days of age. Chicks housing temperature was set at 32 ℃ and 75% relative humidity and maintained for 48 h, subsequently, the birds were exposed to 35 ℃ for 4 h daily five days a week during the experiment period. The immunization program was as follows: immunization against Newcastle disease (NDV) at the age of 7 and 17 days (eye drops), and against Newcastle disease and avian influenza (H9N2) at the age of 7 days (injection under the neck skin).

**Table 6:** Compositions of basal diets (as-fed-basis).

Ingredients	Starter (1–20 days)	Grower (21–35 days)
Yellow corn	53.53	58.47
Soybean meal (44%)	34.90	29.10
Corn gluten meal	5.00	5.00
Vegetable oil	2.50	4.00
Calcium carbonate	1.30	1.15
Di-calcium phosphate	2.10	1.60
Premix*	0.25	0.25
NaCl (salt)	0.25	0.25
DL-methionine	0.17	0.18
L-lysine	0.00	0.00
Calculated composition		
ME (kcal kg^−1^)*	3000	3200
Crude protein	23.00	21.00
Calcium	1.078	0.917
Non-phytate phosphorus	0.513	0.421
Lysine	1.125	1.003
Methionine	0.556	0.538
Methionine + cysteine	0.907	0.890

*Supplied per kilogram of diet: 4000 IU of vitamin A; 80 mg of α-tocopherols acetate; 3 mg of vitamin K3; 2.5 mg of riboflavin; 2.5 mg of thiamin; 25 mg of nicotinic acid; 4 mg of pyridoxine; 800 mg of choline chloride; 0.3 mg of biotin; 1 mg of folic acid; 50 mg of Zn (zinc oxide); 20 mg of Fe; 60 mg of Mn; 12 mg of Cu (copper sulfate-pentahydrate); 0.30 mg of Cobalt; 0.35 mg of Se (sodium selenite); 0.55 g of Mg; 1.3 g of Na (sodium chloride); 0.45 mg of I (calcium iodate); in addition 10 mg of calcium pantothenate acid; 0.02 mg of a coalmine. ** Calculated according to NRC (1994); ME, Metabolizable energy.

### Analysis of additives in the experiment

The multi-strain probiotic mixture was composed of two bacterial strains, *Lactobacillus Acidophilus* (2 × 10^10^ CFU) and *Bacillus Subtilis* (1.8 × 10^9^ CFU). The bacterial strains were supplied from Cairo MIRCEN, Microbiology Culture Collection, Faculty of Agriculture, Ain Shams University, Egypt. Clove essential oil was purchased from Pure Live Company for Investment and Agricultural Development (Cairo, Egypt). Clove essential oil was analyzed by using gas chromatography-mass spectrometry (GC–MS) as described by Juliano et al.^70^, shown in Tables 6 and 7.

**Table 7:** Chemical composition of clove essential oil.

Compound	% of total
Eugenol	69.7
Beta-Caryophyllene	10.8
Eugenyl acetate	15.3
Alpha-Humulene	1.87
Alpha-Copaene	1.18
Alpha-Cubebene	0.65
Caryophyllene oxide	0.34

### Performance and carcass traits

Feed intake for each replicate and the live body weight of each bird were recorded weekly. The deceased chicks were recorded daily to calculate the feed conversion ratio (feed g: body weight gain g). At the end of the experiment (at 35 d), five chickens/groups were randomly chosen for carcass traits evaluation. Dressing percentage ((carcass weight/Live body weight) × 100), liver, abdominal fat, thymus, spleen, and bursa of Fabricius were weighed relatively according to live body weight.

### Nutrient digestibility and digestive enzymes activities

At the end of the experiment (at 35 d), five chickens were randomly picked from each experimental treatment, and each chicken was placed in the digestion cage individually. Chickens were starved for 12 h (to empty the gut canal), then fed on experimental diets for 3 days, and the excreta was collected every 8 h to be analyzed. The feed and excreta were weighed and then dried at 70 °C for 36 h. Dry matter, crude protein, and ether extract contents of the feed and excreta were measured according to AOAC^71^. Three cm of the ileum digesta were taken from five broiler chickens for each group to detect the activity of digestive enzymes (amylase, trypsin, and lipase) as described by Elbaz et al.^12^.

### Serum biochemical indices

Pre-euthanasia blood samples were collected from the jugular vein in tubules without anticoagulant and then centrifuged at 3400× *g* for 9 min (SIGMA 4–15 Lab Centrifuge, Germany) to obtain serum samples, serum was kept at − 20 °C. Total protein, albumin, triglycerides, glucose, total cholesterol (cholesterol, high-density lipoprotein (HDL), and low-density lipoprotein (LDL)), alanine aminotransferase (ALT), and aspartate aminotransferase (AST), were measured calorimetrically (Spectronic 1201, Milton Roy, Ivyland, PA, USA) according to the manufacturer’s instructions. Malondialdehyde (MDA), superoxide dismutase (SOD), and glutathione peroxidase (GPx) were evaluated as an oxidative stress index, as described by Abdel-Moneim Eid et al.^6^. The hemagglutination inhibition (HI) test was used to detect antibody titer against avian influenza virus (AIV) and Newcastle disease virus (NDV) in serum samples, as described by Elbaz et al.^17^ and Abdel-Moneim et al.^6^ respectively. Serum samples were used to detect concentrations of cytokines of interleukin-10 (IL-10), interleukin-6 (IL-6), and tumor necrosis factor-α (TNF-α) using commercial ELISA kits (MyBioSource, San Diego, CA). All examination procedures were performed according to the manufacturer's instructions.

### Ileum microbial and morphometric changes

Segments (~ 3 cm) from the ileum were collected to detect the histomorphological changes (villus height and crypt depth), and stored in a 10% formalin saline solution. Ileal slides (4–5 μm thickness) were cut using a rotary microtome, then examined by optical microscope, as described by Elbaz^23^.

At 35 days of age, directly after slaughter, the contents of the ileum were squeezed into sterile glass bottles to detect microbial changes. One gram of five fresh samples from each experimental group was diluted and plated onto MacConkey agar, deMan, MacConkey, and Rogosa agar to enumerate *Lactobacillus*, *Escherichia coli*, *Enterococcus*, and Total Coliforms, respectively, and the number of microorganisms was converted to log10^72^.

### Statistical analysis

The SPSS software's general linear model (GLM) approach evaluated the collected data in a completely randomized design. Tukey's test was used to determine the significance of mean differences, and all differences were judged significant at *P* < 0.05.

### Ethical approval and informed consent

The experimental procedures were approved by the Experimental Animal Care Committee of Desert Research Center, Egypt (Approval No. 2022–0173), and all protocols were carried out in accordance with guidelines and regulations of the Universal Directive on the Protection of Animals Used for Scientific Purposes. All protocols follow the ARRIVE guidelines for reporting animal research (https://arriveguidelines.org). Euthanasia was done according to the mechanical cervical dislocation method by Koechner Euthanizing Device, as American Veterinary Medical Association-approved recommendations.

## Data Availability

The datasets used and/or analyzed during the current study are available from the corresponding author upon reasonable request.

## Abbreviations

BWG: Body weight gain
AIV: Avian influenza virus
ALT: Alanine aminotransferase
AST: Aspartate aminotransferase
CD: Crypt depth
CON: Basal diet without feed additive
CEO: Basal diet with clove essential oil
CP: Crude protein
DM: Dry matter
EE: Ether extract
EO: Essential oil
FCR: Feed conversion ratio
FI: Feed intake
GPx: Glutathione peroxidase
HDL: High-density lipoprotein
IL-6: Interleukin 6
IL-10: Interleukin 10
LDL: Low-density lipoprotein
MDA: Malondialdehyde
NDV: Newcastle disease virus
PC: Basal diet with probiotics and clove essential oil
PRO: Basal diet with probiotics
SOD: Superoxide dismutase
TNF-α: Tumor necrosis factor-α
VH: Villus height
